# The effectiveness of immersive virtual reality as a student‐centered tool for learning neuroanatomy: A single‐blind randomized controlled trial with physiotherapy students

**DOI:** 10.1002/ase.70068

**Published:** 2025-06-16

**Authors:** Paloma García‐Robles, Esteban Obrero‐Gaitán, Irene Cortés‐Pérez, María del Rocío Ibancos‐Losada, Ángeles Díaz‐Fernández, María Catalina Osuna‐Pérez

**Affiliations:** ^1^ FRATERNIDAD Muprespa Linares Spain; ^2^ Department of Health Sciences University of Jaén Jaén Spain; ^3^ FISIDEC University Center University of Córdoba Cabra Spain

**Keywords:** anatomy education, learning technology, neuroanatomy, physiotherapy students, student‐centered learning, virtual reality

## Abstract

Neuroanatomy is a crucial component of the physiotherapy curriculum, but its complexity can lead to “neurophobia” among students. Immersive virtual reality (IVR) offers an innovative alternative to traditional methods by providing interactive and realistic three‐dimensional images of neurological structures. We aim to evaluate the effectiveness of IVR for learning neuroanatomy and to assess student satisfaction and motivation. We conducted a single‐blind randomized controlled trial with 67 second‐year physiotherapy students. Besides didactic lectures, students were randomly assigned to one of two groups. The first was an IVR group (*n* = 34), in which students studied autonomously using the MetaQuest 2 IVR device with the SharecareYOU VR application (60 min), after having received instructions on how to use it (30 min). The second group (*n* = 33) followed a traditional learning approach using anatomical atlases and textbooks (90 min). Knowledge retention was assessed through an 18‐item questionnaire, while satisfaction, perceived usefulness, and usability of the IVR headset were evaluated using other questionnaires. Evaluations were carried out immediately after the intervention, and four weeks later. The IVR group demonstrated significantly higher knowledge retention overall (*p* < 0.001) as well as in specific areas, such as theoretical understanding, spatial location, and labeling of brain areas (*p* < 0.001). No statistically significant differences were maintained 4 weeks after the intervention (*p* = 0.37). Additionally, IVR students reported greater satisfaction (*p* < 0.001) and found it useful for learning (*p* < 0.001) without adverse events. Our findings show that IVR is a safe supplementary tool that is more effective for enhancing neuroanatomical education in physiotherapy than textbooks and atlases.

## INTRODUCTION

Learning anatomy is basic in the theoretical and clinical training of future healthcare professionals, including physiotherapy students.^1^, ^2^, ^3^ Neuroanatomy is a fundamental pillar of knowledge in physiotherapy as it enables us to understand the pathophysiology and physical therapy approaches for nervous system diseases (stroke, multiple sclerosis, cerebral palsy, or peripheral nerve compressions, among others) or conditions (such as maintained chronic pain by central sensitization process).^4^ Physiotherapy students highlight neuroanatomy as one of the most difficult and challenging subjects in the curriculum due to the complexity of the contents, which gives rise to the phenomenon of *“neurophobia*.”^5^, ^6^ *“Neurophobia”* can be attributed to two factors. On the one hand, students struggle with locating these structures spatially.^7^ It is important to note that understanding neuroanatomy requires the ability to organize and integrate neurological structures in three dimensions. Therefore, students must have visuospatial skills that allow them to visualize, rotate, and transform the two‐dimensional images of books into three‐dimensional structures.^8^ It has been found that these skills are insufficient in low‐performing students.^9^, ^10^ On the other hand, academic stress and anxiety caused by the subjective perception of neuroanatomy being extremely difficult can reduce academic performance, ahead of other factors, such as sleep difficulties (21%–24%) and mental health problems (20%–23%).^11^, ^12^

To date, the most common methods for teaching and learning neuroanatomy are traditional approaches, such as cadaver dissection, two‐dimensional projections or slices of the nervous system during lectures, the use of textbooks and atlases, and the visualization of realistic anatomical plastic models.^13^ These methods are related to “teacher‐centered learning” (TCL) and are characterized by limited interaction between professors and students, which decreases students' interest and motivation in class, ultimately affecting knowledge retention in the medium to long term.^14^, ^15^, ^16^, ^17^ In contrast to the TCL model, “student‐centered learning” (SCL) increases students' interaction and active participation in the teaching‐learning process.^18^, ^19^, ^20^ SCL supports self‐regulated learning (SRL), allowing students to engage actively in their own education using metacognitive, motivational, and behavioral strategies,^21^, ^22^ which are essential for life‐long learning.^23^ Teachers must motivate and provide innovative educational tools that enhance interaction, attention, and knowledge retention of physiotherapy contents, particularly in neuroanatomy.^24^, ^25^

After the COVID‐19 pandemic, the use of technology‐based simulation for teaching purposes has increased, and immersive virtual reality (IVR) is one of the most widely used for teaching and learning anatomy.^26^ IVR devices are the modality of virtual reality that involve high levels of interaction (immersion and presence) between individuals and virtual environments.^27^, ^28^ Through head‐mounted displays (HMD), subjects can visualize highly realistic three‐dimensional environments in 360° that appear to be real.^29^ IVR applications for neuroanatomy show neuroanatomical structures in three dimensions and their spatial relations with others, allowing students to improve their visuospatial abilities and recognize brain areas in different space projections.^30^ Additionally, some IVR devices (such as MetaQuest 2 HMD) allow users to manipulate virtual neuroanatomy images with hand controllers or their own hands (hands‐free interaction),^31^ providing them with an opportunity for “hands‐on” learning, which is fundamental for SCL and SRL.^32^, ^33^ To date, several studies have reported that IVR enhances students' imagination and creativity, while promoting meaningful learning through more enjoyable, engaging, and participatory teaching.^34^ A recent meta‐analysis has shown that IVR is more effective than TCL for knowledge retention of anatomy contents, especially when used as a supplementary tool. Moreover, the students deemed it to be highly useful for learning.^35^

In terms of the physiotherapy curriculum, Lucena‐Antón et al. (2022) suggest that virtual reality could be useful for learning anatomy.^36^ Similarly, Obrero‐Gaitán et al. (2021) propose teaching about neurological disorders and neuroanatomy in physiotherapy using the Leap Motion Controller.^37^ In the only study involving physiotherapy students to date, Kurul et al. (2020) compared an IVR‐based learning model to traditional methods for learning the anatomy of the head and neck,^38^ reporting statistically significant improvements in knowledge acquisition favoring the IVR group. To date, no studies have evaluated the effectiveness of IVR‐based interventions on learning neuroanatomy in physiotherapy students. Therefore, the aim of this study was to assess the effectiveness of an IVR‐based learning intervention on neuroanatomy knowledge acquisition compared with traditional learning through anatomical atlases and textbooks in second‐year physiotherapy students, both immediately after the intervention and at 4‐week follow‐up. Additionally, we sought to analyze students' levels of satisfaction and the usability of this tool for learning neuroanatomy.

## METHODS

### Study design

A single‐blind randomized controlled trial was carried out at the University of Jaén (Jaén, Spain) with second‐year undergraduate physiotherapy students during the 2022–2023 academic year (between April and September 2023). The study protocol was approved by the Ethics Committee of the University of Jaén (ABR.23/5TES). The Declaration of Helsinki guidelines^39^ were followed; all enrolled students participated voluntarily and signed written informed consent.

### Randomization and blinding

The participants who voluntarily consented to participate were randomly assigned to either the experimental group (IVR‐based learning intervention group) or the control group (autonomous study with textbooks and neuroanatomy atlases) using version 3.1 of Epidat software. Randomization was carried out by an external and blinded evaluator.

### Neuroanatomy subject included in the physiotherapy curriculum

Participants were recruited from a group of second‐year undergraduate physiotherapy students enrolled in the subject *“Specific Methods of Intervention in Physiotherapy III”* at the University of Jaén. The contents of this subject include: (1) Basic anatomy, physiology, and pathophysiology of the nervous system; and (2) physiotherapeutic approaches for diagnosing and treating physical, sensorimotor, balance, and functional disorders, as well as central and peripheral nervous system diseases. These conditions include stroke, acquired brain damage, multiple sclerosis, Parkinson's and Alzheimer's disease, spinal cord injury, amyotrophic lateral sclerosis, and neuropathies of the cervical, brachial, lumbar, and sacral plexuses.

### Learning interventions

Seventy‐five students were enrolled in this subject, in which they received theoretical lessons (all students receive a didactic lecture) and practical lessons (students learn in 5 small groups of 14–16 students). This study was carried out in the practical lessons. Firstly, all students of both groups received the same didactic lecture about basic neuroanatomy for 60 min by an expert professor. Later, students in the five groups were randomly assigned (1:1) to the experimental (IVR‐based learning) or the traditional learning intervention (autonomous study using atlases and textbooks) that would last 90 min each.

#### IVR‐based learning intervention

**IMAGE 1:**
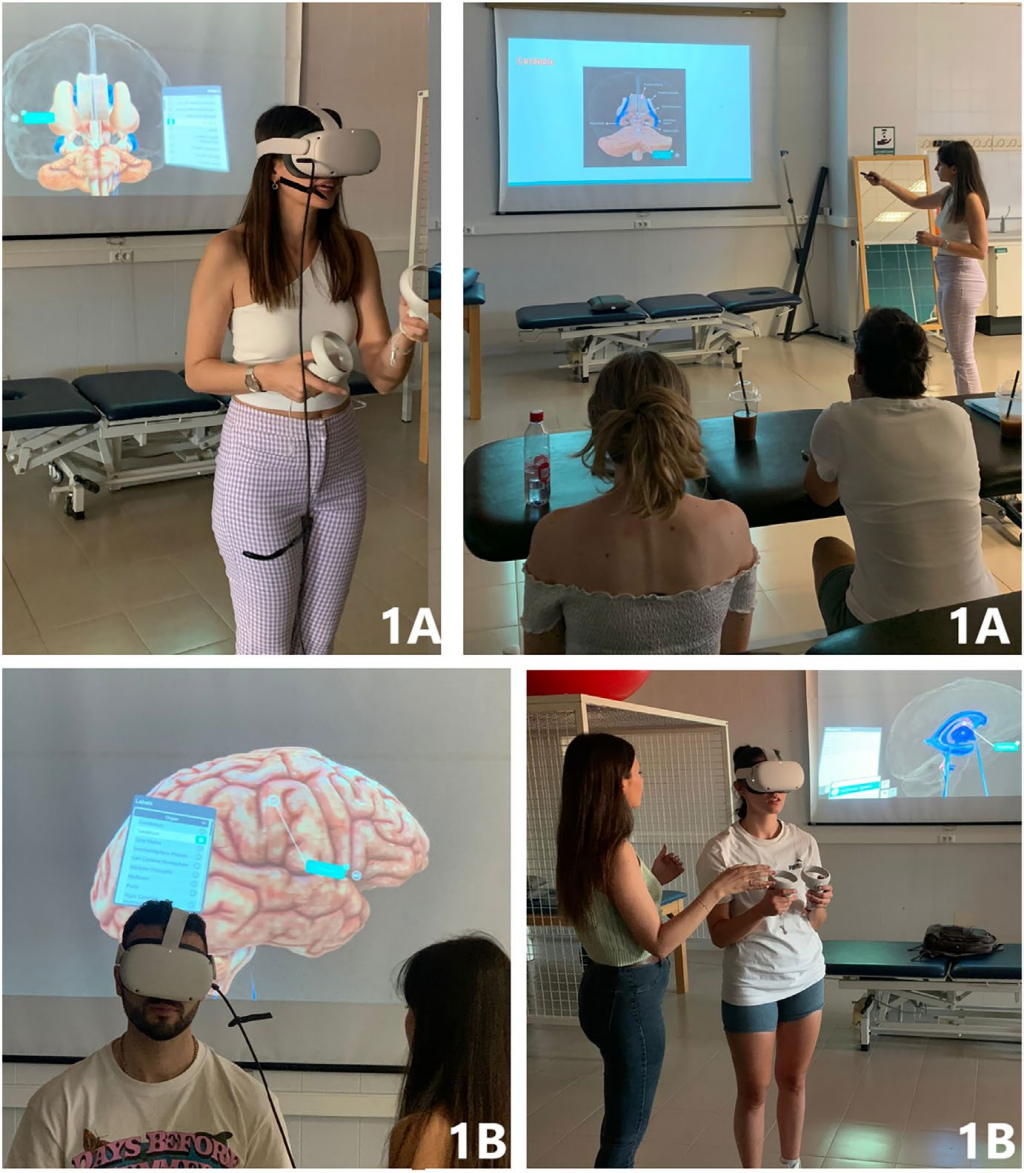
IVR‐based learning intervention: Professor using IVR to explain neuroanatomy (Image 1A). Student interacting with Meta Quest 2 and Sharecare VR to visualize the lateral and third cerebral ventricles (Image 1B).

The IVR‐based learning intervention was carried out in two phases over the 90 min. Firstly, the professor provided general instructions for 30 min on how to use the IVR headset, its controllers, and the anatomy software. In this first phase, the professor projected images of neuroanatomy from Sharecare YOU VR using Meta Quest 2 (through Oculus Casting function of the IVR headset) (Image 1, 1A) onto a large screen. Later, each student was able to practice the aforementioned neuroanatomical content using Sharecare YOU VR by themselves (Image 1, 1B) for 60 min. The professor in charge of the intervention guided and helped the students to locate and name the neuroanatomical structures while they were using the software. This can be carried out using the Oculus Casting function.

The Meta Quest 2 device (Meta, Menlo Park, CA, USA) and the Sharecare YOU VR anatomy app were the hardware and software used, respectively. The Meta Quest 2 headset is a completely autonomous, immersive HMD with a resolution of 1832 × 1920 pixels that supports a 60, 72, and 90 Hz refresh rate. It therefore has all of the components required to be able to operate the immersive application independently while positioning it accurately in a given environment.^40^ The six‐degrees‐of‐freedom motion tracking system integrates four‐camera optical positioning relative to the user's known environment, and its precision is augmented by additional sensors, such as gyroscopes and accelerometers.^41^ The Sharecare YOU VR app delivers medically‐accurate anatomy, physiology, disease, and treatment simulations in impressive 3D detail. It has been designed for interactivity and immersive environments and is currently being used in many medical education institutions worldwide. In addition to displaying 3D anatomical images, Sharecare YOU VR allows users to create custom learning modules, interactive quizzes, and other engaging tools to support their medical curriculum. Of all the anatomical areas available in Sharecare YOU VR, we selected the brain module. This dynamic and completely interactive module showed all the external (cortex and peripheral‐related structures) and internal areas (corpus callosum, thalamus, ventricles, brainstem, among others) with high realism, allowing students to visualize the brain and its structures in different spatial planes. Students could interact with the app using the Meta Quest 2 controllers that allowed the image to be rotated, flipped, maximized, or minimized according to the student's criterion. In addition, it should be noted that the app allowed each of the most relevant brain structures to be named with tags.

#### Traditional learning intervention

Students in the control group received a traditional learning intervention, in which they studied autonomously with textbooks and atlases for 90 min. All students used the same material from (*Prometheus Atlas of Anatomy, Vol. 3 Head, neck and neuroanatomy*) available at the University of Jaén with unlimited free access for students. To guide the autonomous study, each student received a list of neuroanatomical structures that they had to locate and read information about in the textbook or atlas.

### Time points, outcomes, and measurements

#### Time points

Data were collected by a blinded evaluator at three time points. At baseline (T0), researchers compiled sociodemographic information, previous experience with IVR devices and/or video games, experience in tasks that require spatial location, and baseline level of neuroanatomy knowledge. After the intervention (T1), the evaluator measured the level of neuroanatomy knowledge retention in both groups, the level of satisfaction with both interventions and the usefulness of the method. Additionally, the usability and the possible adverse effects associated with IVR exposition were compiled. Finally, 4 weeks after the intervention (T2), the evaluator assessed the level of neuroanatomy knowledge again.

#### Outcomes and measurements

##### Retention or acquisition of neuroanatomy knowledge

The acquisition of neuroanatomy knowledge was assessed using a questionnaire developed by two expert physiotherapy professors with over 10 years of teaching experience in neuroanatomy and neurology. Separate questionnaires were created for each assessment time point (T0, T1, and T2) to evaluate whether students had learned from the intervention in terms of their ability to locate and relate 3D neuroanatomical structures to one another and to other anatomical features. Each questionnaire comprised 18 questions: 6 multiple‐choice questions focused on the spatial location of neuroanatomical structures from different perspectives in various planes (example: In a sagittal section of the brainstem, the pons is located cranial to: (a) corpus callosum, (b) thalamus, (c) medulla oblongata, (d) putamen); 5 multiple‐choice questions assessing theoretical knowledge of the central nervous system structure and function (example: What is the brain structure that connects the right and left cerebral hemispheres? (a) cerebellum, (b) corpus callosum, (c) thalamus, (d) amygdala); and 7 label questions that required students to identify and recognize specific neuroanatomical structures by naming them on a brain slice image (example: A lateral image of the cerebral cortex is provided with an arrow in the fissure between the frontal–parietal and temporal lobes. The student must identify and name that structure: Sylvian fissure). Each correct answer is rewarded with one point, resulting in a total score range of 0–18, where higher scores indicate greater knowledge acquisition. This approach aimed to determine whether students achieved better scores in specific areas based on the educational intervention they received.

##### Level of satisfaction with the intervention

The level of satisfaction was measured using a custom questionnaire comprising 8 items, with scores in a range from 0 (no satisfaction) to 10 (maximum satisfaction). This questionnaire aimed to assess whether the educational intervention method was perceived as appealing, interesting, enjoyable, and whether participants would be inclined to use this method frequently, among other factors.

##### Usefulness for learning neuroanatomy

The level of usefulness for learning was assessed using a custom 7‐item questionnaire, with scores from 0 (lowest score) to 10 (highest score).

##### Usability level of the IVR intervention as an education tool

The level of usability was measured exclusively in the IVR‐based learning group using a custom questionnaire consisting of 9 items for the neuroanatomy software and 6 items for the IVR headset, with scores from 0 (lowest score) to 10 (highest score). The questionnaire evaluated aspects of the application design, including clarity, contrast, realism, and fidelity of the anatomical images in Sharecare YOU VR. For the usability of IVR devices, factors such as ease of use, simplicity, and fluidity of the controllers for interacting with the application were also assessed.

##### Adverse effects

The potential adverse effects associated with the IVR headset were assessed through a custom 19‐item questionnaire, with scores ranging from 0 (no adverse effect experienced) to 10 (strong adverse effect experienced). The following possible adverse effects were considered: fatigue, boredom, drowsiness, headache, disorientation, claustrophobia, nausea, dizziness, difficulty concentrating, general malaise, tired eyes, irritation of the eyes, tearing, dryness of the eyes, eye fatigue, blurry vision, difficulty focusing, double vision, and visual discomfort.

### Statistical analysis

A blinded statistical physiotherapist conducted data handling and analysis using the statistical package for social sciences (SPSS) version 21 (SPSS Inc., Chicago, IL, USA). The results were presented using descriptive statistics, frequencies, and percentages for categorical variables and mean and standard deviation for continuous variables. To study the homogeneity of the groups at the start of the intervention, we used the student t‐test for quantitative variables and the Chi‐squared test for categorical variables. In relation to the knowledge retention analysis, scores for correctly answered questions were expressed as mean, standard deviation (SD), and percentages. Differences in the distribution of the correct responses between IVR and control groups were analyzed with student t‐test for each exam (post‐intervention and at 1‐month follow‐up). Within the IVR group, the impact of the experience in tasks requiring spatial skills was analyzed by contrasting neuroanatomy knowledge post‐intervention and at 1‐month follow‐up between students who reported experience and those who did not. Statistical analysis was conducted considering *p* < 0.05 statistically significant and a level of confidence of 95% (95% CI).

## RESULTS

### Participants

**FIGURE 1:**
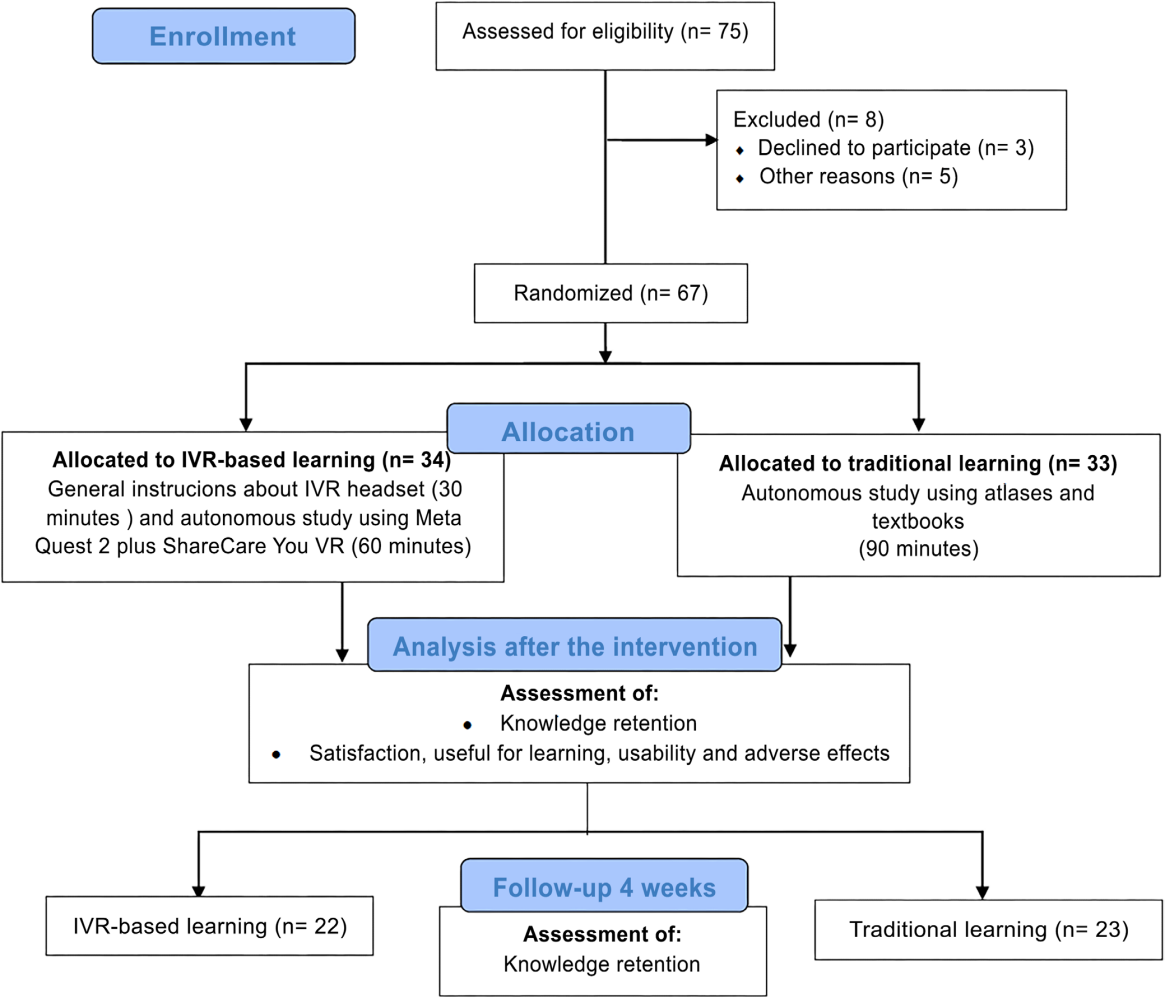
CONSORT flow diagram.

Of the 75 students enrolled in the subject, 67 students (89.3%) participated in this study. Three students declined to participate and 5 were taking part in the Erasmus program abroad when the study began (Figure 1).

The IVR group comprised 34 students (20 males and 14 females) with a mean age of 20.6 ± 1.3 years and with an average grade for knowledge of anatomy from the previous academic year of 7.6 ± 1.1. The control group was composed of 33 students (16 males, 17 females) with a mean age of 21.1 ± 2.2 years and a 7.3 ± 1.5 average grade for baseline knowledge of neuroanatomy. Before the interventions, no statistically significant differences were found between both groups in relation to age (*p* = 0.29), gender (*p* = 0.39), or baseline level of neuroanatomy (*p* = 0.35). Secondly, no statistically significant differences were found between groups in daily use of videogames (*p* = 0.66) and experience in tasks requiring spatial skills (*p* = 0.31). Table 1 shows the baseline characteristics of groups.

**TABLE 1:** Baseline demographics characteristics of students.

Characteristics	IVR group (*n* = 34)	Control group (*n* = 33)	Total cohort (*n* = 67)
Age, mean (±SD)	20.6 (±1.3)	21.1 (±2.2)	20.81 (±1.8)
Male, *n* (%)	20 (59.0)	16 (49.0)	36 (54.0)
Female, *n* (%)	14 (41.0)	17 (51.0)	31 (46.0)
Score in the subject of anatomy from the previous academic year 0–10, mean (SD)	7.6 (±1.1)	7.3 (±1.5)	7.5 (±1.3)
Previous IVR experience, *n* (%)	6 (18.0)	7 (21.0)	13 (19.0)
Daily use of videogames, *n* (%)	12 (35.0)	10 (30.0)	22 (33.0)
Experience in tasks requiring spatial skills, *n* (%)	3 (9.0)	1 (3.0)	4 (6.0)

Abbreviations: IVR, immersive virtual reality; *n*, number of students; SD, standard deviation.

### Retention or acquisition of neuroanatomy knowledge

**FIGURE 2:**
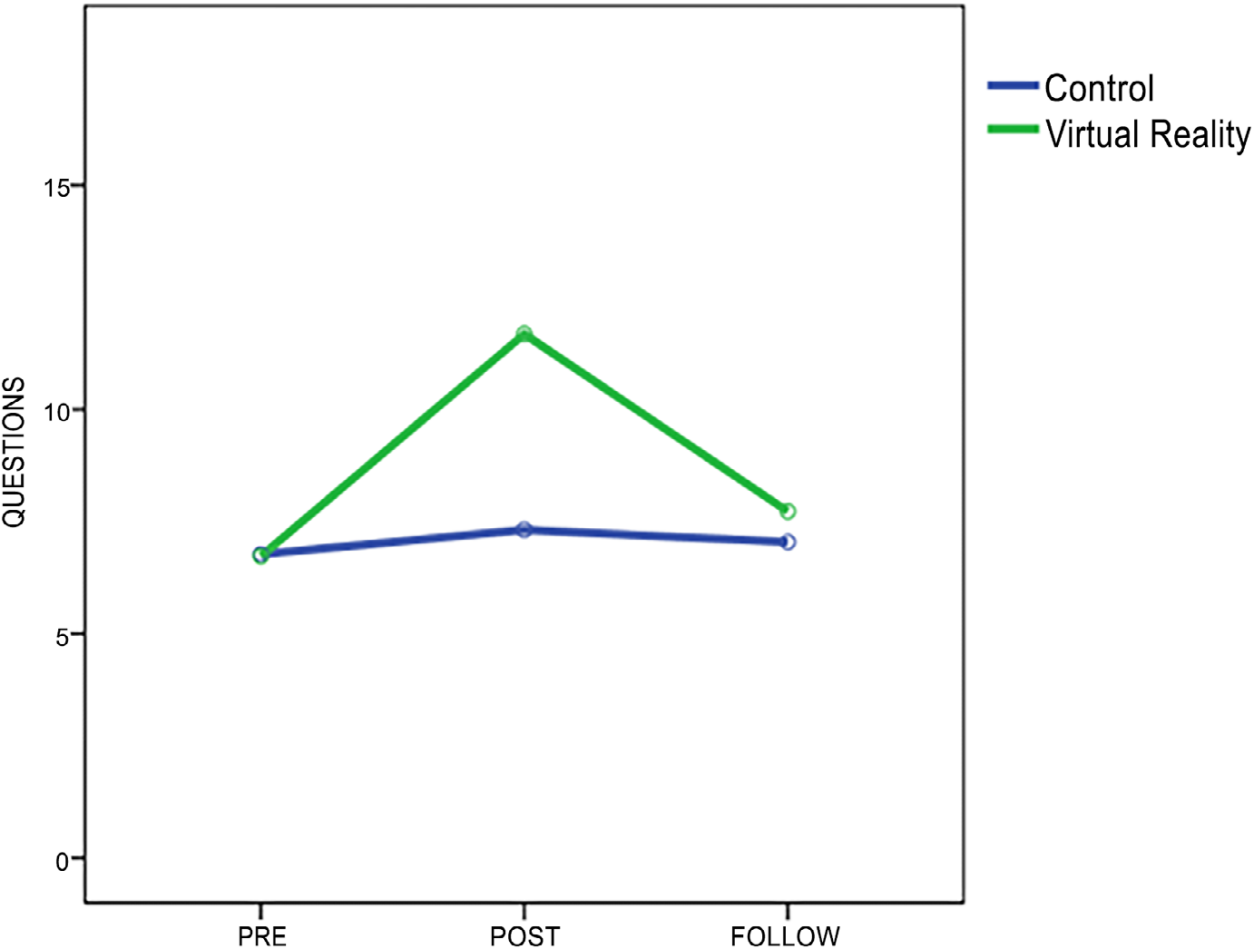
Scores of the 18‐question test for neuroanatomy knowledge in both groups at baseline (pre‐intervention), after the intervention, and 4‐week follow‐up.

Before the interventions, the pre‐test evaluation (Table 2, Figure 2) did not report statistically significant differences between groups in the general level of neuroanatomy knowledge (*p* = 0.66), or in any dimension: spatial orientation (*p* = 0.94), theory (*p* = 0.53), and labels (*p* = 0.68). It demonstrates the homogeneity between groups before the interventions. It is important to note that all students failed the prior knowledge test, regardless of the group to which they belonged.

**TABLE 2:** Effectiveness of virtual reality in reinforcing content.

Time point	Type of questions	IVR group (*n* = 34)		Control group (*n* = 33)		*p*‐Value
		Mean (±SD)	% (0–100)	Mean (±SD)	% (0–100)	
Pre‐test (T0)	General (0–18)	6.5 (±2.1)	36	6.3 (±2.2)	35	0.66
	Spatial (0–6)	2.8 (±1.3)	47	2.8 (±1.35)	47	0.94
	Theory (0–5)	3.1 (±1.2)	62	2.9 (±1.2)	58	0.53
	Label (0–7)	0.6 (±0.7)	9	0.5 (±0.7)	8	0.68
Post‐test (T1)	General (0–18)	11.4 (±2.6)	63	6.5 (±2.6)	36	0.001*
	Spatial (0–6)	3.6 (±1.4)	60	1.9 (±1.2)	31	0.001*
	Theory (0–5)	4.3 (±0.7)	85	3.6 (±1.2)	72	0.001*
	Label (0–7)	3.5 (±1.6)	50	1.1 (±1.3)	15	0.001*
		**IVR group (*n* = 22)**		**Control group (*n* = 23)**		
Follow‐up (T2)	General (0–18)	7.7 (±1.9)	43	7.1 (±2.4)	40	0.37
	Spatial (0–6)	3.3 (±1.1)	55	3.1 (±1.4)	52	0.69
	Theory (0–5)	3.1 (±0.9)	59	2.7 (±0.9)	55	0.16
	Label (0–7)	1.3 (±1.1)	19	1.1 (±1.5)	17	0.67

Abbreviations: IVR, immersive virtual reality; *n*, number of students; SD, standard deviation.

* *p* < 0.05 was considered statistically significant.

At the end of the interventions, statistically significant differences were found between the groups both in the overall result of the post‐test (*p* < 0.001) and in each of the 3 dimensions (*p* < 0.001). The scores of the IVR group in the general test were considerably higher than those of the control group (63% vs. 36%) (Table 2, Figure 2). A lower number of students who studied autonomously using atlases and textbooks passed the theoretical questions (72%) than those in the IVR group (85%) (*p* < 0.001). Scoring on spatial and labeling questions was higher in the IVR group (60% and 50%, respectively) than in the control group (31% and 15%), with statistically significant differences in favor of the IVR group (*p* < 0.001).

Four weeks after the interventions, no statistically significant differences were reported between groups in the general test (*p* = 0.37) (Table 2, Figure 2). The values of both groups were equal in each of the dimensions of the questionnaire and in their overall score. At follow‐up, some participants did not attend, resulting in sample loss. Nonetheless, the groups remained homogeneous in number (*n* = 22 in IVR group and *n* = 23 in control group). Both groups slightly improved their knowledge of neuroanatomy compared with the pre‐test values, but the average level of each group remained low, not exceeding 50% on the knowledge test. Both groups managed to score above 50% in the spatial and theoretical parts. However, the scores in the labeling part of the test were low (19% in the IVR group vs. 17% in the control group).

Within the IVR group, the analysis in relation to previous experience in tasks requiring spatial skills found no significant differences in the overall results of the post‐intervention knowledge test (69% ± 17% of correct answers of those who did report experience vs. 63% ± 14% of those who did not, *p* = 0.53) or after the follow‐up test (39% ± 17% vs. 44% ± 10%; *p* = 507).

### Satisfaction and usefulness for learning

The students in the IVR‐based learning intervention group expressed higher levels of satisfaction (9.6 ± 0.7) than those in the control group (3.4 ± 2.5) (*p* < 0.001). The IVR group scored all satisfaction items (detailed in Table 3) with a value of 9 or more (except for the inverse item: “the method seems boring to me”) while the control group did not exceed an average score of 4.

**TABLE 3:** Results of the satisfaction and utility questionnaires in each group.

Variable	Questions	IVR group (*n* = 34)		Control group (*n* = 33)		*p*‐Value
		Mean	SD	Mean	SD	
Satisfaction	This method is motivating for studying neuroanatomy	9.30	1.23	2.67	2.10	0.001*
	The study method is appealing	9.42	1.03	2.45	2.13	0.001*
	The study method is interesting	9.21	1.86	3.15	2.64	0.001*
	The study method is boring	2.00	3.41	7.76	2.03	0.001*
	The study method is fun	9.21	1.21	1.97	2.18	0.001*
	I would use this study method frequently	9.24	1.11	2.21	1.93	0.001*
	I would use this study method in other subjects	9.45	0.97	2.00	2.09	0.001*
	In general, I am satisfied with the study method used	9.61	0.79	3.21	2.48	0.001*
Usefuleness for learning	This study method allows me to know in which aspects of the subject I need to improve	9.06	1.36	5.67	2.75	0.001*
	This study method could help the autonomous study of the subject	9.48	0.91	5.42	2.42	0.001*
	Its use as a continuous evaluation system for a subject could be useful	9.06	1.32	3.82	2.40	0.001*
	Its use as a final evaluation system for a subject could be useful	8.73	1.66	3.36	2.13	0.001*
	I think it could be useful for anatomical memory	9.61	0.86	5.67	2.59	0.001*
	I think it could be useful for special orientation in anatomy	9.61	0.78	5.18	2.91	0.001*
	In general, I think this study method is useful	9.58	0.79	4.70	2.41	0.001*

*Note*: The scores are expressed in the (0–10) range. Mean and SD (standard deviation).

Abbreviation: SD, standard deviation.

* *p* < 0.05 was considered statistically significant.

Regarding the usefulness of the IVR intervention for learning neuroanatomy, we also found statistically significant differences in all the items evaluated (*p* < 0.001), with a score of 9.6 ± 0.8 vs. 4.7 ± 2.4 in the control group. Table 3 shows that the IVR group gave all the items a score of around 9 (out of 10) while the control group did not score more than 6 for any of the items.

### Design and usability level of the IVR intervention as an education tool

The participants in the IVR group rated both the design and usability of the Meta Quest 2 headset and Sharecare YOU VR very positively (Table 4). In terms of design, the students reported a mean score of 8.8 ± 0.6 points; “realism of the images” and “accurate representation of neuroanatomy” were the highest‐rated items. For usability, the mean score was 8.5 ± 0.4 points with “useful information messages” and “clear instructions” items receiving the highest ratings.

**TABLE 4:** Design of usability of IVR and neuroanatomy app.

	Items	IVR group (*n* = 34)	
		Mean	SD
Design	Clarity of images	8.5	1.6
	Attractiveness of images	9.2	1.1
	Realism of the images	9.5	0.9
	Color contrast	9.4	1
	Label clarity	8.3	2.1
	Adaptation of font size	7.7	2.6
	Intuitive buttons and controls	8.4	1.7
	3D zoom/manipulation quality	8.9	1.3
	Faithful reflection of the anatomy	9.5	0.9
Usability	Ease of use	8.4	1.4
	Fluency (speed)	7.9	2.1
	Intuitive operation	8.2	1.7
	Clear instructions	8.7	1.9
	Intuitive screen transition	8.5	1.7
	Useful informational messages	9	1

*Note*: The ratings are expressed in the (0–10) range. Mean and SD (standard deviation).

Abbreviation: SD, standard deviation.

### Adverse effects after IVR exposition

Overall, no adverse effects were recorded by the participants in the IVR group. The only adverse effects recorded were blurred vision in 1 participant, difficulty focusing on images due to binocular acuity in 1 participant, and somnolence in another.

## DISCUSSION

To date, numerous studies have examined the effectiveness of IVR interventions as an educational approach for learning neuroanatomy among healthcare students, primarily medical students, with promising results in terms of knowledge retention or satisfaction.^35^ However, only one study has assessed the effectiveness of IVR for learning the musculoskeletal structures of the head and neck in physiotherapy students, demonstrating that IVR is superior to traditional interventions in enhancing knowledge retention.^38^ Physiotherapists play a crucial role in the physical rehabilitation of neurological disorders, and a thorough understanding of all neuroanatomical structures involved in these conditions is essential for successful rehabilitation. We hypothesized that IVR‐based learning would be an effective SCL tool to enhance neuroanatomy knowledge among physiotherapy students. Therefore, the aim of our study was to evaluate the effectiveness of an IVR‐based learning intervention in the acquisition of neuroanatomy knowledge (in 3 question modalities: spatial orientation, theory, and labeling of a structure) versus traditional learning methods in physiotherapy students. It was analyzed both immediately after the intervention and four weeks later. Our findings indicated that IVR‐based learning is an effective SCL tool for acquiring neuroanatomy knowledge in the short term. Additionally, physiotherapy students rated this innovative method as satisfying, engaging, enjoyable, and useful for learning compared with studying solely with anatomical atlases or textbooks.

Prior to the intervention, students in both groups failed the pre‐test assessment, thus demonstrating a low level of neuroanatomy knowledge. The students had difficulties in answering theoretical questions correctly (62% in IVR group and 58% in control group), and a very low level of recognizing and labeling brain structures (success rate of 9% in IVR group and 8% in control group). This highlights two important concerns. On the one hand, the “neurophobia” and great difficulty that students report in retaining, recognizing, and identifying brain structures when these images are projected in different positions in traditional atlases. On the other hand, the importance and need to establish new teaching methods to improve learning.

Immediately after the intervention in our study, the IVR group showed a statistically significant improvement in overall retention of neuroanatomy knowledge (63% in IVR group and 36% in control group) and across all three question types (60% vs. 31% in spatial skills questions, 85% vs. 72% in theoretical questions, and 50% vs. 15% in labeling questions, all in favor of the IVR group). Although the anatomical areas studied differed from other studies, our findings align with those reported by Kurul R et al. 2020, who demonstrated that an IVR‐based learning intervention is more effective than traditional lectures in enhancing anatomy knowledge among physiotherapy students.^38^ However, our findings contrast with three previous studies that reported no statistically significant differences between IVR‐based learning and traditional interventions that relied on independent study using textbooks and atlases.^34^, ^42^, ^43^ Another study showed that the major motivation experienced using IVR devices could contribute to higher scores in exams based on knowledge.^44^ An interesting aspect of our findings is that the main benefits provided by IVR devices are related to locating and labeling structures in neuroanatomy, which is where the largest differences between groups were found. Previous experience in tasks requiring spatial skills found no significant differences, although few students reported this experience. It would be important to analyze the impact of individual spatial abilities on the acquisition of neuroanatomy knowledge. Tools like the mental rotation test, for example, could explore whether individual differences affect retention of neuroanatomy when using this particular learning methodology.

Four weeks after the intervention, neuroanatomy knowledge levels declined and were similar in both groups. There were some improvements in the theoretical and spatial orientation questions, but not in labeling. These advances could be attributed to the students' independent study efforts to pass the subject following the intervention. It is important to note that the IVR‐based learning was relatively brief (one session of 90 min), which we believe limited its short‐term impact. However, it is expected that with a greater number of interventions conducted over a longer period and used as a complementary method to traditional teaching, the results obtained could be sustained more effectively over time.

Literature indicates that one of the main reasons for the effectiveness of IVR as a didactic tool is its impact on student satisfaction and motivation. Consistent with previous studies,^45^, ^46^, ^47^ our research demonstrated a high level of satisfaction in students who were allocated to the IVR‐based learning intervention. These students reported that IVR‐based learning is much more motivating, appealing, interesting, and enjoyable than independent study using textbooks and atlases. They were highly satisfied with the methodology and highlighted IVR as a valuable resource for learning neuroanatomy. A key difference between IVR and traditional interventions was that the IVR‐based learning was more useful than atlases in helping students locate, visualize, and spatially recognize brain structures. To ensure effective transfer of neuroanatomical knowledge from classroom learning to clinical practice, it is essential for students to develop the ability to organize brain areas involved in disease and rehabilitation in three dimensions, along with their relationships to adjacent structures.^48^ As our study demonstrates, IVR devices are an excellent option for learning neuroanatomy and integrating its three‐dimensional structure. Furthermore, these systems are considered safe, as the students reported very few adverse effects, which were mainly related to visual disturbances. Notably, other issues commonly mentioned in the literature,^49^ such as headaches or cybersickness, were not reported.

IVR‐based learning is set to become an integral part of the curricula of health disciplines,^50^ which is supported by the results of this study. In line with previous studies,^51^ our findings advocate for the inclusion of IVR devices as a complementary intervention within a SCL approach to teaching neuroanatomy to physiotherapy students. The use of IVR technology, which allows for 360° visualization of neuroanatomical structures, enables students to explore brain areas with greater realism and immersion than traditional atlases, thereby facilitating a deeper and more meaningful understanding of the complexities of the nervous system.^34^, ^43^ Additionally, with an IVR device and immersive software of neuroanatomy, the virtual anatomical model can be manipulated (rotated, maximized, or minimized) in real time, providing a more customized and interactive learning experience that can be tailored to each student's individual needs.^52^ By contrast, traditional textbooks and slide‐based teaching methods can be static and limited in their ability to convey the three‐dimensional complexity of neuroanatomy. Additionally, literature supports the use of IVR devices in education, postulating that it enhances students' imagination, creativity, and meaningful learning through more enjoyable, engaging, and participatory teaching methods.^53^ Several studies in neuroeducation have shown that visual materials promote long‐term memory retention and facilitate the internalization of anatomical knowledge.^26^, ^27^, ^36^, ^54^ A drawback of using these devices is their high cost.^55^ However, in recent years, low‐cost and easy‐to‐use anatomy devices and software, such as the Meta Quest 2 glasses and Sharecare YOU VR application, are becoming more available on the market.

Although findings in this study show relevant implications for learning neuroanatomy among physiotherapy students, some limitations should be taken into account. One of the main limitations was that it was not possible to blind the students or teachers, thus leading to potential bias related to an overestimation of findings. Another limitation was the sample loss in the last assessment at 4 weeks' post‐intervention; some students did not complete the knowledge questionnaire, thus making it difficult to assess the effectiveness of the method in the medium term. It is also important to highlight that the intervention was only implemented in a single teaching session. The use of IVR over a longer period of time is expected to yield better results. Lastly, the assessment tools used were designed ad‐hoc for this study and therefore do not have reliability and validity values for quality criteria.

## CONCLUSION

This is the first study conducted on physiotherapy students to assess the effectiveness of an IVR‐based intervention for learning neuroanatomy. The findings demonstrate that IVR‐based learning is more effective than traditional methods, which rely on independent study using atlases and textbooks, in improving retention of neuroanatomy knowledge and enabling three‐dimensional visualization, recognition, and identification of neuroanatomical areas in the short term. Additionally, the IVR group reported a high level of satisfaction with this methodology, emphasizing that this approach is more motivating, engaging, enjoyable, and useful for learning neuroanatomy than traditional methods. The results of this study could help professors in improving neuroanatomy knowledge among physiotherapy and health sciences students, thereby enhancing the connection between academic curricula and clinical practice. In the future, further studies involving physiotherapy students with larger sample sizes and covering additional anatomical content should be conducted.

## AUTHOR CONTRIBUTIONS

**Paloma García‐Robles:** Conceptualization; investigation; writing – original draft; visualization; software; data curation; formal analysis. **Esteban Obrero‐Gaitán:** Conceptualization; investigation; writing – original draft; methodology; validation; visualization; writing – review and editing; data curation; supervision; project administration. **Irene Cortés‐Pérez:** Investigation; conceptualization; writing – review and editing; visualization; formal analysis; methodology; data curation. **María del Rocío Ibancos‐Losada:** Conceptualization; investigation; writing – review and editing; visualization; data curation. **Ángeles Díaz‐Fernández:** Conceptualization; investigation; writing – review and editing; visualization; data curation. **María Catalina Osuna‐Pérez:** Conceptualization; investigation; writing – original draft; writing – review and editing; visualization; validation; methodology; software; formal analysis; project administration; supervision.

## FUNDING INFORMATION

No external funding was received.

## CONFLICT OF INTEREST STATEMENT

No conflicts of interest are declared by the authors.

## ETHICS STATEMENT

Approval by the Ethics Committee of the University of Jaén (ABR.23/5TES). Written informed consent was obtained from all enrolled participants.

## Data Availability

Request to corresponding author.
